# (*E*)-3-(4-Chloro­phen­yl)-1-(5-hy­droxy-2,2-dimethyl-2*H*-chromen-6-yl)prop-2-en-1-one

**DOI:** 10.1107/S1600536811055255

**Published:** 2012-01-07

**Authors:** Jie Tang, Jin-ying Chen, Ling-qun Jiang

**Affiliations:** a Department of Pharmaceutics, West China of Pharmacy, Sichuan University, Chengdu 610041, People’s Republic of China; b State Key Laboratory of Biotheraphy, West China Hospital, West China Medical Shcool, Sichuan University, Chengdu, Sichuan 610041, People’s Republic of China; c Department of Internal Medicine, Chongqing Medical and Pharmaceutical College, Chongqing, People’s Republic of China

## Abstract

There are two independent mol­ecules in the asymmetric unit of the title compound, C_20_H_17_ClO_3_, each having an *E* configuration about the –C=C– bond. The dihedral angles between the two benzene rings in the two mol­ecules are 7.17 (11) and 9.82 (11)°. In both mol­ecules, the hy­droxy group is involved in an intra­molecular O—H⋯O hydrogen bond.

## Related literature

For the biological activity of chalcones, see: Tran *et al.* (2009 ▶); Rao *et al.* (2004 ▶); Opletalova & Sedivy (1999 ▶); Dimmock *et al.* (1999 ▶). For related structures, see: Boeck *et al.* (2006 ▶); Jasinski *et al.* (2009 ▶); Wang & Yang (2011 ▶). For the synthesis of a related compound, see: Krohn *et al.* (2002 ▶).
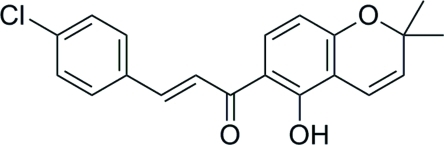


## Experimental

### 

#### Crystal data

C_20_H_17_ClO_3_
*M* *_r_* = 340.79
Monoclinic, 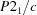

*a* = 11.0641 (7) Å
*b* = 9.8255 (5) Å
*c* = 30.7834 (14) Å
β = 91.177 (4)°
*V* = 3345.8 (3) Å^3^
*Z* = 8
Mo *K*α radiation
μ = 0.24 mm^−1^
*T* = 293 K
0.30 × 0.25 × 0.20 mm

#### Data collection

Oxford Diffraction Xcalibur Eos diffractometer
Absorption correction: multi-scan (*CrysAlis PRO*; Oxford Diffraction, 2010 ▶) *T*_min_ = 0.979, *T*_max_ = 1.0
15168 measured reflections
6836 independent reflections
4040 reflections with *I* > 2σ(*I*)
*R*_int_ = 0.023

#### Refinement

*R*[*F* ^2^ > 2σ(*F* ^2^)] = 0.054
*wR*(*F* ^2^) = 0.145
*S* = 1.01
6836 reflections
439 parameters
H-atom parameters constrained
Δρ_max_ = 0.17 e Å^−3^
Δρ_min_ = −0.32 e Å^−3^

### 

Data collection: *CrysAlis PRO* (Oxford Diffraction, 2010 ▶); cell refinement: *CrysAlis PRO*; data reduction: *CrysAlis PRO*; program(s) used to solve structure: *SHELXS97* (Sheldrick, 2008 ▶); program(s) used to refine structure: *SHELXL97* (Sheldrick, 2008 ▶); molecular graphics: *OLEX2* (Dolomanov *et al.*, 2009 ▶); software used to prepare material for publication: *OLEX2*.

## Supplementary Material

Crystal structure: contains datablock(s) I, global. DOI: 10.1107/S1600536811055255/lh5397sup1.cif

Structure factors: contains datablock(s) I. DOI: 10.1107/S1600536811055255/lh5397Isup2.hkl

Supplementary material file. DOI: 10.1107/S1600536811055255/lh5397Isup3.cml

Additional supplementary materials:  crystallographic information; 3D view; checkCIF report

## Figures and Tables

**Table 1:** Hydrogen-bond geometry (Å, °)

*D*—H⋯*A*	*D*—H	H⋯*A*	*D*⋯*A*	*D*—H⋯*A*
O2—H2⋯O3	0.82	1.82	2.537 (2)	146
O5—H5*A*⋯O6	0.82	1.81	2.532 (2)	147

**Fig. 1.**
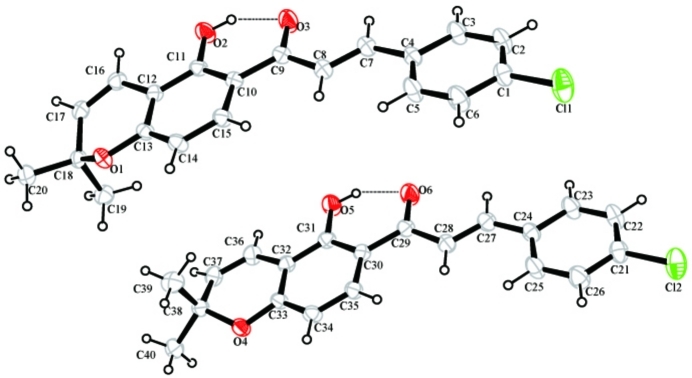
The two independent molecules of (I) with 30% probability displacement ellipsoid. Dashed lines denote intermolecular hydrogen bonds.

## supplementary crystallographic information

### Comment

Chalcones (1,3-diaryl-2-propen-1-ones) are stucturally simple compounds of the flavonoid family which display an impressive array of biological properties such as anticancer (Rao *et al.*, 2004; Tran *et al.*, 2009), antileishmanial (Boeck *et al.*, 2006), antifungal activity (Opletalova & Sedivy, 1999) and antioxidant activity (Dimmock *et al.*, 1999). Chalcones can be easily obtained from the adol condensation of aromatic aldehydes and aromatic ketones. The crystal structure of related compounds:(*E*)-1-(5-Hydroxy-2,2-dimethl-2*H*-chromen-6-yl)-3-(3,4,5-trimethoxyphenyl)prop-2-en-1-one (Wang *et al.*, 2011) and (2E)-1-(2-Bromoppheny)-3-(3,4,5-trimethoxypheny)prop-2-en-1-one (Jasinski *et al.*, 2009) have been reported recently. The crystal structure of the title compound (I) is reported in this paper.

The asymmetric unit of (I) consists of two crystallographically independent molecules (Fig. 1). The bond distances are normal and are comparable with a closely related structure (Wang *et al.*, 2011). Each molecule of (I) exists in an E configuration with respect to the C7═C8 double bond. The dihedral angles between the two benzene rings in each molecule are 7.17 (11)° [C21—C26/C30—C35] and 9.82 (11)° [C1—C6/C10—C15]. In both independent molecules, the hydroxy group is invovled in an intramolecular O—H···O hydrogen bond.

### Experimental

1-(5-hydroxy-2,2-dimethyl-2*H*-chromen-6-yl)ethanone(2.182 g,10 mmol), 4-chlorobenzaldehyde(1.962 g, 10 mmol) in ethanal KOH (20% *w*/*v* aqueous solution) and the mixture was stirred at 273 K for 10 h. The the crude product was recrystallized from ethanol to give (I). Single crystals suitable for X-ray structure determination were grown by slow evaporation of a diethyl ether solution of (I) at room temperature.

### Refinement

H atoms were positioned geometrically (C—H = 0.93–0.96 Å, O—H = 0.86 Å) and constrained to ride on their parent atoms in a riding-model approximation, with U*_iso_*(H) = 1.2*_eq_*(C) or 1.5U*_eq_*(C_methyl_,*O*).

### Crystal data

**Table:**

C_20_H_17_ClO_3_	*F*(000) = 1424
*M**_r_* = 340.79	*D*_x_ = 1.353 Mg m^−^^3^
Monoclinic, *P*2_1_/*c*	Mo *K*α radiation, λ = 0.7107 Å
Hall symbol: -P 2ybc	Cell parameters from 3974 reflections
*a* = 11.0641 (7) Å	θ = 2.9–29.2°
*b* = 9.8255 (5) Å	µ = 0.24 mm^−^^1^
*c* = 30.7834 (14) Å	*T* = 293 K
β = 91.177 (4)°	Block, yellow
*V* = 3345.8 (3) Å^3^	0.30 × 0.25 × 0.20 mm
*Z* = 8	

### Data collection

**Table:**

Oxford Diffraction Xcalibur Eos diffractometer	6836 independent reflections
Radiation source: fine-focus sealed tube	4040 reflections with *I* > 2σ(*I*)
graphite	*R*_int_ = 0.023
Detector resolution: 16.0874 pixels mm^-1^	θ_max_ = 26.4°, θ_min_ = 2.9°
ω scans	*h* = −11→13
Absorption correction: multi-scan (CrysAlis Pro; Oxford Diffraction, 2010)	*k* = −10→12
*T*_min_ = 0.979, *T*_max_ = 1.0	*l* = −38→38
15168 measured reflections	

### Refinement

**Table:**

Refinement on *F*^2^	Primary atom site location: structure-invariant direct methods
Least-squares matrix: full	Secondary atom site location: difference Fourier map
*R*[*F*^2^ > 2σ(*F*^2^)] = 0.054	Hydrogen site location: inferred from neighbouring sites
*wR*(*F*^2^) = 0.145	H-atom parameters constrained
*S* = 1.01	*w* = 1/[σ^2^(*F*_o_^2^) + (0.0503*P*)^2^ + 0.9761*P*] where *P* = (*F*_o_^2^ + 2*F*_c_^2^)/3
6836 reflections	(Δ/σ)_max_ < 0.001
439 parameters	Δρ_max_ = 0.17 e Å^−^^3^
0 restraints	Δρ_min_ = −0.32 e Å^−^^3^

### Special details

**Table:**

Geometry. All e.s.d.'s (except the e.s.d. in the dihedral angle between two l.s. planes) are estimated using the full covariance matrix. The cell e.s.d.'s are taken into account individually in the estimation of e.s.d.'s in distances, angles and torsion angles; correlations between e.s.d.'s in cell parameters are only used when they are defined by crystal symmetry. An approximate (isotropic) treatment of cell e.s.d.'s is used for estimating e.s.d.'s involving l.s. planes.
Refinement. Refinement of *F*^2^ against ALL reflections. The weighted *R*-factor *wR* and goodness of fit *S* are based on *F*^2^, conventional *R*-factors *R* are based on *F*, with *F* set to zero for negative *F*^2^. The threshold expression of *F*^2^ > σ(*F*^2^) is used only for calculating *R*-factors(gt) *etc*. and is not relevant to the choice of reflections for refinement. *R*-factors based on *F*^2^ are statistically about twice as large as those based on *F*, and *R*- factors based on ALL data will be even larger.

### Fractional atomic coordinates and isotropic or equivalent isotropic displacement parameters (Å^2^)

**Table:**

	*x*	*y*	*z*	*U*_iso_*/*U*_eq_	
Cl1	0.17697 (7)	0.68585 (9)	0.12791 (2)	0.0832 (3)	
Cl2	0.67696 (7)	0.18823 (9)	0.13224 (2)	0.0867 (3)	
O1	0.43040 (13)	1.34774 (15)	−0.21832 (4)	0.0446 (4)	
O2	0.55742 (17)	1.50025 (16)	−0.07889 (5)	0.0591 (5)	
H2	0.5497	1.4826	−0.0531	0.089*	
O3	0.49425 (19)	1.36093 (17)	−0.01377 (5)	0.0685 (6)	
O4	0.92657 (15)	0.83125 (15)	−0.21812 (4)	0.0535 (4)	
O5	1.05691 (17)	0.98927 (17)	−0.07956 (5)	0.0630 (5)	
H5A	1.0497	0.9722	−0.0537	0.094*	
O6	0.98945 (19)	0.85726 (18)	−0.01371 (5)	0.0702 (6)	
C1	0.2403 (2)	0.8166 (3)	0.09786 (7)	0.0516 (6)	
C2	0.3173 (2)	0.9068 (3)	0.11760 (7)	0.0601 (7)	
H2A	0.3368	0.8980	0.1470	0.072*	
C3	0.3659 (2)	1.0107 (3)	0.09376 (7)	0.0559 (7)	
H3	0.4183	1.0722	0.1073	0.067*	
C4	0.3383 (2)	1.0259 (2)	0.04984 (6)	0.0440 (5)	
C5	0.2598 (2)	0.9333 (3)	0.03089 (7)	0.0603 (7)	
H5	0.2401	0.9410	0.0015	0.072*	
C6	0.2099 (2)	0.8296 (3)	0.05478 (7)	0.0645 (8)	
H6	0.1561	0.7688	0.0417	0.077*	
C7	0.3899 (2)	1.1385 (2)	0.02538 (7)	0.0484 (6)	
H7	0.4321	1.2035	0.0416	0.058*	
C8	0.3838 (2)	1.1588 (2)	−0.01696 (7)	0.0501 (6)	
H8	0.3432	1.0958	−0.0345	0.060*	
C9	0.4395 (2)	1.2785 (2)	−0.03733 (7)	0.0467 (6)	
C10	0.4317 (2)	1.3002 (2)	−0.08425 (6)	0.0411 (5)	
C11	0.4949 (2)	1.4095 (2)	−0.10305 (6)	0.0409 (5)	
C12	0.4962 (2)	1.4278 (2)	−0.14818 (6)	0.0391 (5)	
C13	0.43243 (19)	1.3360 (2)	−0.17420 (6)	0.0379 (5)	
C14	0.3628 (2)	1.2328 (2)	−0.15658 (6)	0.0445 (6)	
H14	0.3160	1.1766	−0.1745	0.053*	
C15	0.3640 (2)	1.2151 (2)	−0.11228 (6)	0.0439 (5)	
H15	0.3187	1.1449	−0.1005	0.053*	
C16	0.5543 (2)	1.5420 (2)	−0.16934 (7)	0.0481 (6)	
H16	0.5829	1.6151	−0.1529	0.058*	
C17	0.5664 (2)	1.5415 (2)	−0.21202 (7)	0.0469 (6)	
H17	0.5973	1.6179	−0.2257	0.056*	
C18	0.5310 (2)	1.4197 (2)	−0.23871 (6)	0.0409 (5)	
C19	0.6367 (2)	1.3223 (2)	−0.24194 (7)	0.0521 (6)	
H19A	0.6611	1.2928	−0.2133	0.078*	
H19C	0.7030	1.3675	−0.2554	0.078*	
H19B	0.6128	1.2448	−0.2591	0.078*	
C20	0.4809 (2)	1.4566 (2)	−0.28360 (7)	0.0533 (6)	
H20A	0.4528	1.3756	−0.2981	0.080*	
H20C	0.5433	1.4981	−0.3003	0.080*	
H20B	0.4149	1.5191	−0.2808	0.080*	
C21	0.7398 (2)	0.3173 (3)	0.10131 (7)	0.0540 (6)	
C22	0.8127 (2)	0.4117 (3)	0.12071 (7)	0.0647 (7)	
H22	0.8297	0.4070	0.1504	0.078*	
C23	0.8612 (2)	0.5144 (3)	0.09617 (7)	0.0601 (7)	
H23	0.9112	0.5787	0.1095	0.072*	
C24	0.8371 (2)	0.5239 (2)	0.05185 (7)	0.0467 (6)	
C25	0.7623 (3)	0.4268 (3)	0.03335 (7)	0.0627 (7)	
H25	0.7445	0.4308	0.0037	0.075*	
C26	0.7131 (3)	0.3235 (3)	0.05785 (8)	0.0672 (8)	
H26	0.6625	0.2591	0.0449	0.081*	
C27	0.8875 (2)	0.6353 (2)	0.02659 (7)	0.0498 (6)	
H27	0.9296	0.7014	0.0423	0.060*	
C28	0.8806 (2)	0.6535 (2)	−0.01581 (7)	0.0514 (6)	
H28	0.8402	0.5894	−0.0329	0.062*	
C29	0.9352 (2)	0.7731 (2)	−0.03680 (7)	0.0478 (6)	
C30	0.9275 (2)	0.7921 (2)	−0.08387 (6)	0.0423 (5)	
C31	0.9920 (2)	0.8995 (2)	−0.10336 (7)	0.0440 (5)	
C32	0.9926 (2)	0.9147 (2)	−0.14846 (7)	0.0428 (5)	
C33	0.9251 (2)	0.8254 (2)	−0.17399 (6)	0.0423 (5)	
C34	0.8573 (2)	0.7219 (2)	−0.15562 (7)	0.0482 (6)	
H34	0.8108	0.6644	−0.1732	0.058*	
C35	0.8599 (2)	0.7056 (2)	−0.11134 (7)	0.0468 (6)	
H35	0.8156	0.6353	−0.0992	0.056*	
C36	1.0660 (2)	1.0162 (2)	−0.17048 (8)	0.0536 (6)	
H36	1.1210	1.0689	−0.1546	0.064*	
C37	1.0540 (2)	1.0329 (2)	−0.21284 (7)	0.0516 (6)	
H37	1.1061	1.0921	−0.2268	0.062*	
C38	0.9593 (2)	0.9595 (2)	−0.23919 (7)	0.0463 (6)	
C39	0.8463 (2)	1.0461 (3)	−0.24342 (8)	0.0666 (7)	
H39B	0.7852	0.9969	−0.2595	0.100*	
H39C	0.8648	1.1289	−0.2584	0.100*	
H39A	0.8173	1.0671	−0.2150	0.100*	
C40	1.0025 (3)	0.9172 (3)	−0.28349 (7)	0.0618 (7)	
H40B	1.0746	0.8635	−0.2802	0.093*	
H40C	1.0195	0.9968	−0.3004	0.093*	
H40A	0.9408	0.8644	−0.2980	0.093*	

### Atomic displacement parameters (Å^2^)

**Table:**

	*U*^11^	*U*^22^	*U*^33^	*U*^12^	*U*^13^	*U*^23^
Cl1	0.0787 (6)	0.1037 (6)	0.0670 (4)	−0.0313 (5)	0.0003 (4)	0.0278 (4)
Cl2	0.0782 (6)	0.1026 (6)	0.0797 (5)	−0.0145 (5)	0.0132 (4)	0.0333 (4)
O1	0.0441 (9)	0.0561 (10)	0.0337 (8)	−0.0075 (8)	0.0009 (6)	−0.0009 (7)
O2	0.0831 (14)	0.0533 (10)	0.0405 (8)	−0.0213 (9)	−0.0060 (9)	−0.0060 (8)
O3	0.1044 (16)	0.0582 (11)	0.0422 (9)	−0.0197 (11)	−0.0150 (9)	0.0015 (8)
O4	0.0721 (12)	0.0492 (10)	0.0393 (8)	−0.0154 (9)	0.0071 (8)	−0.0031 (7)
O5	0.0772 (14)	0.0610 (11)	0.0502 (9)	−0.0267 (10)	−0.0132 (9)	−0.0005 (8)
O6	0.0999 (16)	0.0619 (11)	0.0480 (10)	−0.0190 (11)	−0.0197 (10)	0.0024 (8)
C1	0.0450 (15)	0.0673 (16)	0.0428 (13)	−0.0030 (13)	0.0042 (10)	0.0080 (11)
C2	0.0624 (18)	0.0832 (19)	0.0345 (12)	−0.0096 (15)	−0.0052 (11)	0.0073 (12)
C3	0.0566 (17)	0.0716 (17)	0.0391 (13)	−0.0122 (14)	−0.0081 (11)	0.0006 (11)
C4	0.0433 (14)	0.0540 (14)	0.0349 (11)	0.0028 (12)	0.0027 (10)	−0.0011 (10)
C5	0.0686 (19)	0.0784 (19)	0.0335 (12)	−0.0145 (15)	−0.0063 (11)	0.0046 (12)
C6	0.0683 (19)	0.0797 (19)	0.0452 (14)	−0.0226 (16)	−0.0072 (12)	0.0014 (13)
C7	0.0508 (15)	0.0553 (15)	0.0392 (12)	0.0003 (12)	0.0000 (10)	−0.0002 (10)
C8	0.0603 (17)	0.0491 (14)	0.0408 (13)	0.0001 (13)	0.0013 (11)	−0.0001 (10)
C9	0.0553 (16)	0.0445 (13)	0.0402 (12)	0.0027 (12)	−0.0015 (11)	−0.0032 (10)
C10	0.0477 (14)	0.0390 (12)	0.0366 (11)	0.0005 (11)	0.0021 (10)	−0.0033 (9)
C11	0.0452 (14)	0.0377 (12)	0.0395 (12)	−0.0006 (11)	−0.0041 (10)	−0.0063 (9)
C12	0.0411 (13)	0.0376 (12)	0.0386 (11)	0.0015 (10)	−0.0007 (9)	−0.0018 (9)
C13	0.0343 (12)	0.0441 (13)	0.0355 (11)	0.0030 (10)	0.0022 (9)	−0.0034 (9)
C14	0.0432 (14)	0.0500 (14)	0.0404 (12)	−0.0108 (11)	0.0026 (10)	−0.0076 (10)
C15	0.0476 (15)	0.0432 (13)	0.0411 (12)	−0.0072 (11)	0.0073 (10)	−0.0018 (10)
C16	0.0571 (16)	0.0360 (12)	0.0510 (14)	−0.0045 (12)	−0.0049 (11)	−0.0018 (10)
C17	0.0550 (16)	0.0368 (13)	0.0490 (13)	−0.0016 (11)	0.0012 (11)	0.0077 (10)
C18	0.0443 (14)	0.0405 (12)	0.0382 (11)	−0.0007 (11)	0.0049 (9)	0.0050 (9)
C19	0.0473 (15)	0.0524 (15)	0.0568 (14)	0.0042 (12)	0.0057 (11)	0.0022 (11)
C20	0.0617 (17)	0.0558 (15)	0.0424 (13)	0.0031 (13)	0.0019 (11)	0.0068 (11)
C21	0.0438 (15)	0.0686 (17)	0.0499 (14)	0.0008 (13)	0.0075 (11)	0.0114 (12)
C22	0.0641 (19)	0.092 (2)	0.0375 (13)	−0.0050 (17)	−0.0026 (12)	0.0111 (13)
C23	0.0592 (18)	0.0791 (19)	0.0416 (13)	−0.0120 (15)	−0.0078 (11)	0.0018 (12)
C24	0.0404 (14)	0.0597 (15)	0.0401 (12)	0.0030 (12)	0.0029 (10)	−0.0010 (11)
C25	0.0721 (19)	0.0786 (19)	0.0371 (13)	−0.0127 (16)	−0.0021 (12)	0.0018 (12)
C26	0.072 (2)	0.0755 (19)	0.0543 (16)	−0.0215 (16)	0.0037 (13)	−0.0004 (13)
C27	0.0474 (15)	0.0563 (15)	0.0458 (13)	−0.0008 (12)	−0.0012 (11)	−0.0012 (11)
C28	0.0606 (17)	0.0499 (14)	0.0437 (13)	−0.0044 (13)	0.0015 (11)	−0.0003 (10)
C29	0.0530 (16)	0.0455 (14)	0.0445 (13)	0.0019 (12)	−0.0050 (11)	−0.0018 (10)
C30	0.0445 (14)	0.0411 (13)	0.0413 (12)	0.0000 (11)	0.0000 (10)	−0.0025 (10)
C31	0.0420 (14)	0.0421 (13)	0.0477 (13)	−0.0043 (11)	−0.0040 (10)	−0.0030 (10)
C32	0.0413 (14)	0.0412 (13)	0.0459 (12)	−0.0028 (11)	0.0018 (10)	−0.0004 (10)
C33	0.0456 (14)	0.0446 (13)	0.0369 (12)	−0.0017 (11)	0.0044 (10)	−0.0045 (10)
C34	0.0539 (16)	0.0458 (14)	0.0449 (13)	−0.0144 (12)	0.0037 (11)	−0.0081 (10)
C35	0.0520 (15)	0.0451 (13)	0.0437 (13)	−0.0082 (12)	0.0063 (10)	−0.0002 (10)
C36	0.0506 (16)	0.0522 (15)	0.0577 (15)	−0.0150 (13)	−0.0043 (11)	0.0048 (12)
C37	0.0469 (15)	0.0505 (14)	0.0576 (15)	−0.0085 (12)	0.0052 (11)	0.0104 (11)
C38	0.0502 (15)	0.0448 (13)	0.0441 (12)	−0.0023 (12)	0.0058 (10)	0.0018 (10)
C39	0.0501 (17)	0.0729 (19)	0.0768 (18)	0.0113 (15)	−0.0021 (13)	−0.0082 (14)
C40	0.077 (2)	0.0615 (17)	0.0480 (14)	0.0043 (15)	0.0155 (13)	0.0049 (12)

### Geometric parameters (Å, °)

**Table:**

Cl1—C1	1.739 (2)	C19—H19A	0.9600
Cl2—C21	1.739 (2)	C19—H19C	0.9600
O1—C13	1.363 (2)	C19—H19B	0.9600
O1—C18	1.471 (2)	C20—H20A	0.9600
O2—H2	0.8200	C20—H20C	0.9600
O2—C11	1.343 (2)	C20—H20B	0.9600
O3—C9	1.237 (3)	C21—C22	1.360 (3)
O4—C33	1.360 (2)	C21—C26	1.365 (3)
O4—C38	1.466 (3)	C22—H22	0.9300
O5—H5A	0.8200	C22—C23	1.376 (3)
O5—C31	1.345 (2)	C23—H23	0.9300
O6—C29	1.237 (3)	C23—C24	1.388 (3)
C1—C2	1.363 (3)	C24—C25	1.379 (3)
C1—C6	1.367 (3)	C24—C27	1.460 (3)
C2—H2A	0.9300	C25—H25	0.9300
C2—C3	1.374 (3)	C25—C26	1.382 (3)
C3—H3	0.9300	C26—H26	0.9300
C3—C4	1.388 (3)	C27—H27	0.9300
C4—C5	1.379 (3)	C27—C28	1.318 (3)
C4—C7	1.461 (3)	C28—H28	0.9300
C5—H5	0.9300	C28—C29	1.477 (3)
C5—C6	1.379 (3)	C29—C30	1.462 (3)
C6—H6	0.9300	C30—C31	1.415 (3)
C7—H7	0.9300	C30—C35	1.404 (3)
C7—C8	1.319 (3)	C31—C32	1.397 (3)
C8—H8	0.9300	C32—C33	1.386 (3)
C8—C9	1.474 (3)	C32—C36	1.461 (3)
C9—C10	1.461 (3)	C33—C34	1.390 (3)
C10—C11	1.412 (3)	C34—H34	0.9300
C10—C15	1.407 (3)	C34—C35	1.372 (3)
C11—C12	1.401 (3)	C35—H35	0.9300
C12—C13	1.389 (3)	C36—H36	0.9300
C12—C16	1.454 (3)	C36—C37	1.318 (3)
C13—C14	1.390 (3)	C37—H37	0.9300
C14—H14	0.9300	C37—C38	1.497 (3)
C14—C15	1.375 (3)	C38—C39	1.515 (3)
C15—H15	0.9300	C38—C40	1.513 (3)
C16—H16	0.9300	C39—H39B	0.9600
C16—C17	1.323 (3)	C39—H39C	0.9600
C17—H17	0.9300	C39—H39A	0.9600
C17—C18	1.498 (3)	C40—H40B	0.9600
C18—C19	1.516 (3)	C40—H40C	0.9600
C18—C20	1.522 (3)	C40—H40A	0.9600
			
O1—C13—C12	121.14 (19)	C18—C20—H20C	109.5
O1—C13—C14	116.92 (18)	C18—C20—H20B	109.5
O1—C18—C17	109.95 (17)	C19—C18—C20	110.97 (18)
O1—C18—C19	108.40 (17)	H19A—C19—H19C	109.5
O1—C18—C20	103.70 (17)	H19A—C19—H19B	109.5
O2—C11—C10	122.11 (18)	H19C—C19—H19B	109.5
O2—C11—C12	116.62 (19)	H20A—C20—H20C	109.5
O3—C9—C8	118.49 (19)	H20A—C20—H20B	109.5
O3—C9—C10	120.2 (2)	H20C—C20—H20B	109.5
O4—C33—C32	121.5 (2)	C21—C22—H22	120.2
O4—C33—C34	117.05 (19)	C21—C22—C23	119.6 (2)
O4—C38—C37	110.53 (18)	C21—C26—C25	119.2 (2)
O4—C38—C39	108.0 (2)	C21—C26—H26	120.4
O4—C38—C40	104.35 (18)	C22—C21—Cl2	119.83 (19)
O5—C31—C30	121.79 (19)	C22—C21—C26	120.9 (2)
O5—C31—C32	117.2 (2)	C22—C23—H23	119.3
O6—C29—C28	118.6 (2)	C22—C23—C24	121.3 (2)
O6—C29—C30	120.2 (2)	C23—C22—H22	120.2
C1—C2—H2A	120.2	C23—C24—C27	120.4 (2)
C1—C2—C3	119.5 (2)	C24—C23—H23	119.3
C1—C6—C5	119.5 (2)	C24—C25—H25	119.3
C1—C6—H6	120.2	C24—C25—C26	121.5 (2)
C2—C1—Cl1	119.86 (18)	C24—C27—H27	116.1
C2—C1—C6	120.7 (2)	C25—C24—C23	117.5 (2)
C2—C3—H3	119.4	C25—C24—C27	122.1 (2)
C2—C3—C4	121.3 (2)	C25—C26—H26	120.4
C3—C2—H2A	120.2	C26—C21—Cl2	119.2 (2)
C3—C4—C7	120.2 (2)	C26—C25—H25	119.3
C4—C3—H3	119.4	C27—C28—H28	119.2
C4—C5—H5	119.4	C27—C28—C29	121.7 (2)
C4—C7—H7	116.2	C28—C27—C24	127.9 (2)
C5—C4—C3	117.7 (2)	C28—C27—H27	116.1
C5—C4—C7	122.10 (19)	C29—C28—H28	119.2
C5—C6—H6	120.2	C30—C29—C28	121.3 (2)
C6—C1—Cl1	119.4 (2)	C30—C35—H35	119.1
C6—C5—C4	121.2 (2)	C31—O5—H5A	109.5
C6—C5—H5	119.4	C31—C30—C29	119.70 (19)
C7—C8—H8	119.1	C31—C32—C36	123.3 (2)
C7—C8—C9	121.9 (2)	C32—C31—C30	120.97 (19)
C8—C7—C4	127.7 (2)	C32—C33—C34	121.43 (19)
C8—C7—H7	116.2	C32—C36—H36	120.0
C9—C8—H8	119.1	C33—O4—C38	119.11 (16)
C10—C9—C8	121.3 (2)	C33—C32—C31	118.8 (2)
C10—C15—H15	119.1	C33—C32—C36	117.84 (19)
C11—O2—H2	109.5	C33—C34—H34	120.4
C11—C10—C9	119.75 (19)	C34—C35—C30	121.8 (2)
C11—C12—C16	123.84 (19)	C34—C35—H35	119.1
C12—C11—C10	121.26 (18)	C35—C30—C29	122.7 (2)
C12—C13—C14	121.85 (18)	C35—C30—C31	117.61 (19)
C12—C16—H16	120.1	C35—C34—C33	119.3 (2)
C13—O1—C18	117.98 (15)	C35—C34—H34	120.4
C13—C12—C11	118.22 (19)	C36—C37—H37	119.0
C13—C12—C16	117.84 (18)	C36—C37—C38	122.0 (2)
C13—C14—H14	120.5	C37—C36—C32	119.9 (2)
C14—C15—C10	121.9 (2)	C37—C36—H36	120.0
C14—C15—H15	119.1	C37—C38—C39	110.1 (2)
C15—C10—C9	122.7 (2)	C37—C38—C40	113.0 (2)
C15—C10—C11	117.56 (18)	C38—C37—H37	119.0
C15—C14—C13	119.01 (19)	C38—C39—H39B	109.5
C15—C14—H14	120.5	C38—C39—H39C	109.5
C16—C17—H17	119.4	C38—C39—H39A	109.5
C16—C17—C18	121.1 (2)	C38—C40—H40B	109.5
C17—C16—C12	119.8 (2)	C38—C40—H40C	109.5
C17—C16—H16	120.1	C38—C40—H40A	109.5
C17—C18—C19	110.31 (19)	H39B—C39—H39C	109.5
C17—C18—C20	113.22 (18)	H39B—C39—H39A	109.5
C18—C17—H17	119.4	H39C—C39—H39A	109.5
C18—C19—H19A	109.5	C40—C38—C39	110.63 (19)
C18—C19—H19C	109.5	H40B—C40—H40C	109.5
C18—C19—H19B	109.5	H40B—C40—H40A	109.5
C18—C20—H20A	109.5	H40C—C40—H40A	109.5

### Hydrogen-bond geometry (Å, °)

**Table:**

*D*—H···*A*	*D*—H	H···*A*	*D*···*A*	*D*—H···*A*
O2—H2···O3	0.82	1.82	2.537 (2)	146.
O5—H5A···O6	0.82	1.81	2.532 (2)	147.
